# DNA methylation aging clocks: challenges and recommendations

**DOI:** 10.1186/s13059-019-1824-y

**Published:** 2019-11-25

**Authors:** Christopher G. Bell, Robert Lowe, Peter D. Adams, Andrea A. Baccarelli, Stephan Beck, Jordana T. Bell, Brock C. Christensen, Vadim N. Gladyshev, Bastiaan T. Heijmans, Steve Horvath, Trey Ideker, Jean-Pierre J. Issa, Karl T. Kelsey, Riccardo E. Marioni, Wolf Reik, Caroline L. Relton, Leonard C. Schalkwyk, Andrew E. Teschendorff, Wolfgang Wagner, Kang Zhang, Vardhman K. Rakyan

**Affiliations:** 1 William Harvey Research Institute, Barts and The London School of Medicine and Dentistry, Queen Mary University of London, London, UK; 2 The Blizard Institute, Barts and The London School of Medicine and Dentistry, Queen Mary University of London, London, UK; 3 Sanford Burnham Prebys Medical Discovery Institute, La Jolla, CA USA; 4 Beatson Institute for Cancer Research and University of Glasgow, Glasgow, UK; 5 Department of Environmental Health Sciences, Mailman School of Public Health, Columbia University, New York, NY USA; 6 Medical Genomics, Paul O’Gorman Building, UCL Cancer Institute, University College London, London, UK; 7 Department of Twin Research and Genetic Epidemiology, King’s College London, London, UK; 8 Department of Epidemiology, Geisel School of Medicine, Dartmouth College, Lebanon, NH USA; 9 Department of Molecular and Systems Biology, Geisel School of Medicine, Dartmouth College, Lebanon, NH USA; 10 Department of Community and Family Medicine, Geisel School of Medicine, Dartmouth College, Lebanon, NH USA; 11 Division of Genetics, Department of Medicine, Brigham and Women’s Hospital and Harvard Medical School, Boston, MA USA; 12 Molecular Epidemiology, Department of Biomedical Data Sciences, Leiden University Medical Center, Leiden, the Netherlands; 13 Department of Human Genetics, Gonda Research Center, David Geffen School of Medicine, Los Angeles, CA USA; 14 Department of Biostatistics, School of Public Health, University of California–Los Angeles, Los Angeles, CA USA; 15 San Diego Center for Systems Biology, University of California–San Diego, San Diego, CA USA; 16 Fels Institute for Cancer Research, Lewis Katz School of Medicine, Temple University, Philadelphia, PA USA; 17 Department of Epidemiology, Brown University, Providence, RI USA; 18 Department of Pathology and Laboratory Medicine, Brown University, Providence, RI USA; 19 Centre for Genomic and Experimental Medicine, Institute of Genetics and Molecular Medicine, University of Edinburgh, Edinburgh, UK; 20 Centre for Cognitive Ageing and Cognitive Epidemiology, University of Edinburgh, Edinburgh, UK; 21 Epigenetics Programme, The Babraham Institute, Cambridge, UK; 22 The Wellcome Trust Sanger Institute, Cambridge, UK; 23 Medical Research Council Integrative Epidemiology Unit (MRC IEU), School of Social and Community Medicine, University of Bristol, Bristol, UK; 24 School of Biological Sciences, University of Essex, Colchester, UK; 25 CAS Key Laboratory of Computational Biology, CAS-MPG Partner Institute for Computational Biology, Shanghai Institute of Nutrition and Health, Shanghai Institutes for Biological Sciences, University of Chinese Academy of Sciences, Chinese Academy of Sciences, 320 Yue Yang Road, Shanghai, 200031 China; 26 UCL Cancer Institute, Paul O’Gorman Building, University College London, 72 Huntley Street, London, WC1E 6BT UK; 27 Helmholtz-Institute for Biomedical Engineering, Stem Cell Biology and Cellular Engineering, RWTH Aachen Faculty of Medicine, Aachen, Germany; 28 Faculty of Medicine, Macau University of Science and Technology, Taipa, Macau

## Abstract

Epigenetic clocks comprise a set of CpG sites whose DNA methylation levels measure subject age. These clocks are acknowledged as a highly accurate molecular correlate of chronological age in humans and other vertebrates. Also, extensive research is aimed at their potential to quantify biological aging rates and test longevity or rejuvenating interventions. Here, we discuss key challenges to understand clock mechanisms and biomarker utility. This requires dissecting the drivers and regulators of age-related changes in single-cell, tissue- and disease-specific models, as well as exploring other epigenomic marks, longitudinal and diverse population studies, and non-human models. We also highlight important ethical issues in forensic age determination and predicting the trajectory of biological aging in an individual.

## Introduction

A key question in biology is to understand why and how we age. Alongside this, the unprecedented gain in the average lifespan in humans, since the mid-twentieth century, has dramatically increased both the number of older people and their proportion in the population. This demographic phenomenon is changing our societal make-up, from only ~130 million being 65 years or older (~5% of the world population) in 1950, to a predicted ~1.6 billion people (~17%) by 2050 [1]. However, the success in reducing mortality has not been matched with a reduction in chronic disease [2]. This leads to the undesirable outcome of many years of this prolonged lifespan being spent in ill health, with an associated massive health care burden. Increasing the productivity and reducing the disease affliction in these extended years7 would be clearly beneficial for both the individual and society [2, 3]. This aim of maximizing the “healthspan” [2] makes obtaining accurate measures of aging-related pathology essential, to gauge its speed, decipher the changes that occur, and potentially unlock how aging acts as a disease risk factor [4]. There is considerable population variation in the rate at which people visibly age [5] as well as become impaired by age-related frailty and disease [2]. Measurement of this relative “biological” aging [2] may allow pre-emptive targeted health-promoting interventions, perhaps in a personalized and disease-specific fashion. It would also aid in testing interventions that attempt to modulate the aging process [6].

The cellular and molecular hallmarks of aging include changes associated with cell senescence, dysregulated nutrient sensing, and stem cell exhaustion, among others [6]. Therefore, many biological measures, such as p16^ink4a^ tissue levels, circulating CRP, creatinine, and fasting glucose, as well as telomere length all correlate with aging [6–8]. In this decade, we have discovered the remarkable power of epigenetic changes to estimate an individual’s age [9, 10]. Epigenetics encapsulates the chemical modifications and packaging of the genome that influence or indicate its activity [11], with strict definitions requiring inheritance through mitotic cell division [12]. Observations of age impacting on this mechanism have been reported for more than 50 years [13–16] and suggested a role in age-related disease [17]. However, the association between epigenetic modifications and age became most starkly apparent with the arrival of the first high-throughput arrays measuring DNA methylation [18–20]. These high-resolution data enabled the construction of extremely accurate age estimators, termed “Epigenetic” or “DNA methylation clocks” [21–25]. Subsequently, these clocks were reported to capture aspects of biological aging and its associated morbidity and mortality [26–29]. DNA methylation (5′methylcytosine, 5mC) is the most common DNA modification and predominantly occurs at cytosines in a CpG dinucleotide context in differentiated mammalian cells. The stability of 5mC in biological samples, even from long-term stored DNA, brings large-scale data availability, for use in subsequent high-throughput analysis.

In this paper, we discuss the scientific challenges that the fascinating discovery of “DNA methylation clocks” has brought into focus. We provide recommendations and suggest future experiments required to dissect the strengths and weaknesses of this important biomarker, in order to probe its biological significance, cellular mechanics, and epidemiological potential. We do not review in depth the background history and current state of the clocks themselves; we refer readers to recent excellent reviews for this information [9, 10]. Instead, the purpose is forward-focused, i.e., to define the current issues, to suggest what will aid unlocking future potential, and to further explore and define any functionality, with the hopeful long-term benefit of increasing the “healthspan.”

Here, we define a “DNA methylation clock” as an estimator built from epigenetic DNA methylation marks that are strongly correlated (*r* ≥ 0.8 [9]) with chronological age or time, which can accurately quantify an age-related phenotype or outcome, or both. These DNA methylation clocks are generally built with a supervised machine learning method, such as a penalized regression (e.g., lasso or elastic net) trained against chronological age to identify an informative and sparse predictive set of CpGs [9, 10]. The residual, or error from chronological age, is used as a marker for biological age of an individual [9, 10]. The age-related phenotype or outcome may be disease, mortality, clinical measures of “frailty,” or cellular phenotypes, including the mitotic age (the total number of lifetime cell divisions of a tissue [30, 31]).

It is evident, even from our initial observations so far, that the aging-related epigenetic modifications captured by DNA methylation clocks are pervasive and indicative of genomic, cell biology, and tissue changes occurring over the life-course. These molecular alterations may bring a high-resolution and precise understanding of age-related pathology and physiology.

## Challenge 1

### Delineation of the chronological and biological components of DNA methylation clocks

#### Current knowledge

DNA methylation-derived epigenetic clocks are currently better in estimating actual chronological age than transcriptomic and proteomic data, or telomere length [7]. However, it was recognized that some variability in these initial clocks’ age estimation existed, which was identified to be a measure capturing individual variation in biological age. Age acceleration, defined as the difference between this epigenetically measured age and the actual chronological age, was associated with mortality [26] and other age-related phenotypes or diseases [32–39].

Of the first-reported clocks, the Hannum et al. clock was trained and tested on blood-derived DNA [23]. It comprises 71 CpG selected from the Illumina 450k array that strongly capture changes in chronological age, which is partly driven by age-related shifts in blood cell composition [23]. The Horvath clock was constructed across multiple tissues, including the blood data from Hannum et al., as a potential “pan-tissue” master clock of chronological age, and focused on capturing shared changes, independent of tissue type [24]. It included 353 CpGs that were present on the earlier generation Illumina 27k array. These differences in training sets led to some conflicting findings between reported associations [7, 29, 40].

Aging leads to epigenetic alterations, including changes in DNA methylation, through both multiple distinct and intersecting age-related mechanisms [6, 41]. Many DNA methylation aging clocks have now been derived, and due to their individual strengths and weaknesses, explicit reference must be made to the specific clock employed (see further in “Challenge 2”). Captured age-related epigenetic variation can be firstly split into intrinsic, or intra-cellular, and extrinsic, or broadly within-tissue and external, aspects of the aging process [27]. The former is a surrogate readout of multiple cellular and genomic processes, including possible deterioration of mechanisms involved in maintaining the epigenome, while the latter includes age-related cell proportion changes within a tissue. While these first clocks are markers capturing these effects to a greater or lesser extent [42], both can predict all-cause mortality at a population, but not individual level, even after correcting for known risk factors [27]. To investigate biological age more directly, clocks have also been trained on age-related and disease phenotypes in combination with chronological age, such as the “PhenoAge” DNA methylation clock that incorporates nine age-related biochemical measures [43]. Cigarette smoking, a significant disease-related factor, is observed to strongly drive mortality-associated predictive DNA methylation changes [44]. However, these tobacco-related methylation changes do not influence the Horvath or Hannum et al. clocks, but are captured in “PhenoAge” [9]. Of note, a very recently constructed mortality predictive DNA methylation clock, termed “GrimAge,” directly incorporates smoking-related changes through an estimate of “pack-years” smoking. This clock also includes certain plasma protein levels estimated by DNA methylation, and this leads to an even stronger prediction of both lifespan and healthspan [45].

#### Current uncertainty

**Fig. 1:**
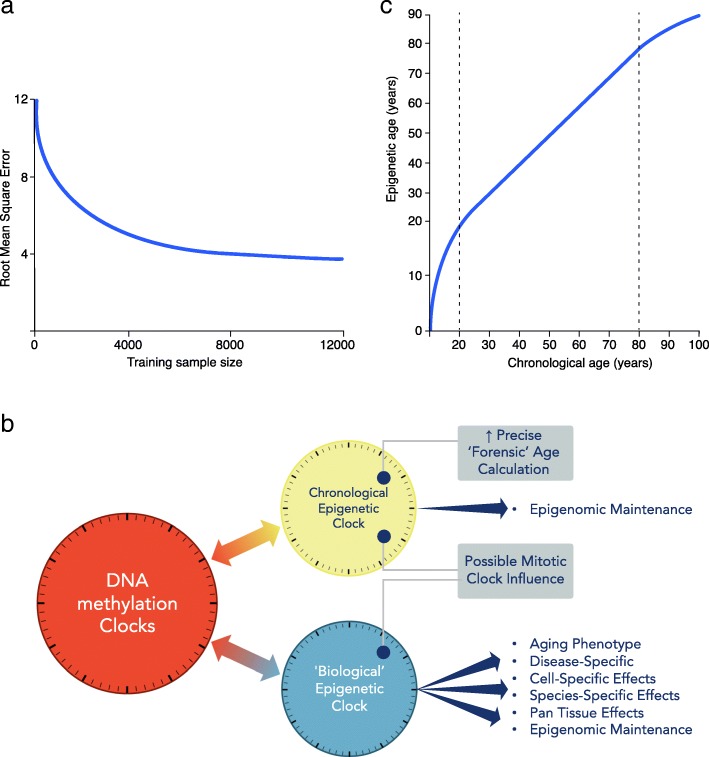
**a** Chronological age estimation error. With increasing training sample size, improved measurement of chronological age is expected, even using current array data (adapted from Zhang et al. [46]). *y*-axis: root mean square error (RMSE) of the predicted age. **b** DNA methylation clocks contain both chronological and biological information. The relative proportions of each will depend on the CpG probes employed in the construction of the clock. Therefore, there are multiple clocks that can be deconvoluted from aging-related epigenetic changes. Moving forward, more precise chronological (forensic age clock) and biological clocks, specific for particular diseases, informative of health or disease state need to be defined and separated. **c** Epigenetic age trajectory. Epigenetic age is not linear over the life course. Chronological age in years (*x*-axis) and epigenetic age in years (*y*-axis)

The first DNA methylation clocks devised were found to be useful for estimating actual age, as well as capturing associations with biological aspects of aging. Data gathered from these early clocks can still be exploited for both these chronological and biological measures. However, now this duality has been recognized, we can attempt to improve our assessment of these two characteristics. Specialized clocks are likely to be more powerful for accurate age prediction or to capture specific biological aging-related functional deterioration or disease-related predictions [45]. How far these two distinct uses can be separated into discrete clocks and improved for their specific role is presently unknown. However, clearly if the DNA methylation clock measurement of actual age was perfect, the loss of any variability removes the window where biological aging associations can be made [46]. Empirical calculations estimate that near-perfect forensic age determination may be possible with large enough sample size, even with current DNA methylation array platforms (see Fig. 1a) [46], although this statistically derived view that chronological clocks can approach extreme precision is not held by all in the field.

Each DNA methylation clock that is constructed is unique to its method of calibration [47], indicating the importance of tissue/s employed, number of samples, and statistical methodology. Clearly, small sample sizes are more susceptible to multiple aging-related confounders, measurement errors, and imperfect statistical predictions. Even when clocks are directly trained on actual chronological age, the strong influence of age-related biological processes may skew the CpGs selected for the clock, underscoring the importance of an appropriate population of sample donors. Furthermore, as discussed in “Challenge 3,” Zhang et al. recently highlighted the impact of not only sample size but also cell type correction, in heterogeneous cell type-derived DNA, on improving chronological age prediction [46].

For “Biological” clocks, another obvious area of uncertainty is that there is not one measure or “gold standard” of biological aging [6, 7, 41, 48]. This phenomenon encompasses a wide range of age-associated changes from the merely visible to disease-risk related. To understand how aging may be characterized by chronological and biological age-related epigenetic changes, we need more detailed understanding of what mechanisms may be underlying these observations. There is no evidence that the Horvath or Hannum et al. clock CpGs are enriched for functionality over and above the promoter-focused arrays from which they were constructed. Furthermore, the clocks have shown variability in their ability to capture measures of mitotic age, such as telomere length [9, 49], due to their differing training models. In general, epigenetic aging is distinct from senescence-mediated aging and is not prevented by telomerase expression [50–52]. A recent DNA methylation telomere clock identified that although this clock was trained on telomere length, it more strongly reflected cell replication and, moreover, associated with aging-related phenotypes more strongly than telomere length itself [53].

#### Future experiments and recommendations

Understanding the chronological and biological drivers of these DNA methylation clocks will require them to be teased apart as much as possible (see Fig. 1b). The clear separation between these two factors, down to specific sets of CpGs, would lead to more powerful specialized clocks and distinct mechanistic studies.

To obtain the most precise estimate of actual age and quantification of its robustness from easily assessable DNA requires appropriately powered large-scale DNA methylation analyses. This is especially the case if this measure is to be used as a legal measure of human age [54–57]. Testing across the range of routinely collected DNA samples will be needed, such as those gathered from peripheral blood or buccal swabs, but also other sources of DNA, such as hair root, skin, and other tissues. However, this is currently only likely to be tractable in data derived from peripheral blood, as these are available at large scale. For the other tissues, the approach is likely to be insufficiently powered in the intermediate future. Specific CpGs will be selected to construct clocks for high-precision forensic age estimation, when chronological age is not known or disputed. They will employ those CpGs that are the most robust and accurate for particular tissues and their constituent cell types [58]. We will need to define the influence of genetic variation and environmental factors on these measures. Accumulating this knowledge of the various DNA methylation clocks will guide their future legal or forensic application [59].

The biological aging component captured by epigenetic age acceleration consists of a large range of drivers, including tissue-specific, cellular aging pathology, stochastic deterioration, and disease-related factors. As mentioned, there is no single measure of biological age; therefore, specific components of aging biology should be focused on and interrogated. This includes aging-related biological pathways involving, for example, mTOR, IGF-1, and p53 [6], as well as epigenomic aspects including the polycomb repressive complex, TET/DNMT levels, and H3K36 methylation [60, 61] (see Table 1). This refined analysis could bring new molecular mechanistic insight to the aging process.

**Table 1:** Major biological and analytic issues with epigenetic DNA methylation clocks

Significant issue	Current problem	Potential solutions/advances
Biological age measures. No single measure or “gold standard” of biological age is likely to be possible	Simultaneously measuring multiple contributing biological processes that are changing with age	Focused analysis on specific components of aging biology and/or specific age-related diseases
Prediction versus mechanistic insight	Predictors are by design not optimal for mechanistic insight but are nevertheless used	Separate prediction (using sparse CpG sets) from mechanistic studies (based on whole DNA methylome/integrated epigenome)
Age-associated changes in non-dividing cells	Uncertainty over mechanism and current ability to dissect apart intrinsic (intracellular) from extrinsic (whole tissue) changes	Analyze aging in single cells. Also, determine whether construction of single-cell clocks is possible
Confusion between epigenetic correlations of aging and the aging process itself	Aging is a complex multi-systemic process. Specific evidence is required that the epigenetic changes observed in DNA methylation clocks are driving the aging process itself	To reduce confusion for those outside the epigenomics field, publications need to be clear that epigenetic observations usually only represent a biomarker of aging
Bias of DNA methylation clock training sets	Uncertainty of the contribution of deviation between predicted and actual age to biological aging or prediction error. Clocks trained on small samples are prone to confounding by cell composition	Larger studies, as well as increasingly focused tissue/disease-relevant clocks and cell type-specific information
Contribution to DNA methylation clock signals of cell type proportions and rare cells and/or clonality	Uncertainty whether cell type deconvolution increases or decreases biomarker power for different diseases	Refined single-cell analysis to separate tissue cell proportional changes from intrinsic cell changes for specific diseases. Also, purified cell type analyses and further refinement of cell type deconvolution methodology
Pan-tissue aging changes	Separation of true pan-tissue changes from cell proportion changes	Single-cell analysis to identify cell proportion changes from individual cell changes. Also, purified cell type and statistical cell type deconvolution analyses
Aging-related increased variability in DNA methylation versus directional changes	Difficult to deconvolute intrinsic from extrinsic changes in heterogeneous cell type-derived DNA, as well as to delineate technical from biological variation	Single-cell analysis to differentiate cell proportion from individual cell changes. Use of multiple technical and statistical methodologies to dissect origin of variability, including deep-targeted BS-seq and third-generation sequencing
Construction of a clock at the single-cell level	Currently technically challenging, particularly due to missing data in each individual cell	Imputation may be helpful, but ultimately improvements in single-cell analysis will be required
Identification of disease-related changes	Uncertainty whether capturing the most disease relevant changes	Improved methodology: latest array increased enhancer CpGs focus—improved high-throughput power to identify tissue-specific and disease-specific loci. Also, increased deep BS sequencing
Disease mechanism is unknown	Role of aging-related epigenomic deterioration contributing to age-related disease pathology is not appreciated	Discovery of disease-related mechanisms through disease-relevant cell and tissue-type epigenomic analysis of aging-related changes
Regulatory role of DNA methylation is more complex than classical models	Complex interplay between transcription factors and epigenomic factors impacts on outcome within different functional loci (promoters, enhancers, insulators, transcribed regions, etc.)	Detailed experimental evidence within specific genetic loci and in disease-relevant cell types, including appropriate disease stressors, to infer potential repressive and/or activating roles
Differentiation between DNA methylation loss due to reduction in active processes required for maintenance, or active enzymatic-driven loss	Firm evidence required in appropriate cell types of decay without cell division. May be more prevalent at dynamic enhancer regions. Neuronal cells have high post-mitotic expression of DNMTs and TETs plus high 5-hydroxymethylcytosine (5hmC)	Detailed models studying DNMT and TET expression in disease-appropriate cell types. Assaying the specific products of TET activity, such as 5hmC
Functionality of DNA methylation changes is often assumed	Crossing statistical significance thresholds does not infer function. Statistical differences between quantitative and categorial measures	Acknowledged functional evidence deficiency in results and that further integrated disease-relevant tissue genomic/epigenomic/transcriptomic analyses in appropriate models are required
Low reproducibility of DNA methylation clocks in model organisms reduces the utility of published clocks	Technical issue because of the reliance on low-depth sequencing due to the lack of available commercial DNA methylation arrays in non-human species	Higher-depth base-resolution sequencing studies are required to improve portability of DNA methylation clocks between experiments. Also, new methodology robust to stochastic missing data
Aging DNA methylation sites are only partially conserved among different mammalian species	Reduced insights to be made from comparative studies	Integrative whole epigenome analyses to identify common mechanistic processes
Role of DNA methylation and rare modifications, such as 5-hydroxymethylation (5hmC) in specific functional loci, such as enhancers	Large-scale base-resolution analyses currently performed using bisulfite conversion. This does not differentiate between 5hmC and 5mC	Oxidative bisulfite sequencing and new methodologies, such as a non-destructive DNA deaminase, and third-generation direct modification analysis
Interconnected role of DNA modifications and chromatin modifications	Unknown directionality and causative effects of cross talk between these different epigenomic modifications	In vitro, organoid, and model organism evaluation of epigenetic machinery with age. Integration of DNA modifications, histone post-translational marks, and transcriptional data into a single integrated aging model
Population variation in DNA methylation clock measures	Genetic variation may be influencing clock measures directly, or impacting on relevant causative factors, such as inflammation and immunological aging	Integrating genetic effectors into clock and age-related measures, including haplotypic information. This will also lead to insights into causal or mechanistic pathways
Many different DNA methylation clock models	Many available clocks and ad hoc application and interpretation of results can result in suboptimal robustness of findings	Systematic evaluation of methods with a priori assumptions about the meaning of associations of various measures
Forensic use of DNA methylation clocks to determine legal age	Robustness of DNA methylation clocks across populations, tissues, and environments is unknown. Furthermore, the impact of acute and chronic inflammatory processes needs assessment	Assess variability by the analysis of large, diverse, and well-powered datasets in the range of tissues likely to be employed (whole blood, buccal cells, etc.)
DNA methylation clocks as a de facto measure of an individual’s “health”	Associations with biological aging are cross-sectional and epidemiological. Accuracy within an individual and in other populations the clock is not derived from is unknown	Longitudinal studies required to assess clock changes within an individual over time. Requires appropriately powered studies across diverse populations. Re-commercialization—public must be protected by provision of accurate data regarding estimate/error rates

Tissue-specific clocks have the potential to be highly clinically useful as prognostic and diagnostic markers of disease, as discussed in the following “Challenge 2.” However, we should not forget about the potentially intriguing insights into aging biology that could be identified by modifications that occur across all tissue types in the body, or pan-tissue changes [62]. Strong outlier candidates for pan-tissue changes identified to date should be further evaluated, such as DNA hypermethylation in *ELOVL2*, as well as looking for novel aging-related chromatin marks. To confirm any consistency of changes across tissue types will ultimately require large-scale and detailed evaluation of single cell types over time. These points, along with distinguishing the effects of cell type composition-driven variation in DNA methylation, are discussed in more detail in “Challenge 3” and “Challenge 5” and will all contribute significantly to improved accuracy and understanding of clock-related measures.

## Challenge 2

### Functional characterization of tissue-specific and disease-specific clocks

#### Current knowledge

“Biological age” is a large umbrella term for multiple age-related phenotypes and disease processes. Observed disease-related DNA methylation changes will represent the tissue specificity of the particular epigenome, indicative of the mixture of cell types present, as well as the associated organ-specific pathology. While the “sum of parts” Horvath pan-tissue clock is extremely useful, it is unlikely to correspond perfectly to each tissue-specific component. Still, disease-specific aging clocks in easily accessible tissues have high potential for clinical utility as disease-specific monitors and disease risk calculators. However, recognition and identification of those changes that are in fact tissue-specific [63] may also enable improved markers of both chronological and biological age. The benefit of bespoke clocks is seen in the recent “Skin-Blood” clock, devised due to poor performance of the Horvath clock in estimating advanced age in Hutchinson-Gilford progeria syndrome, potentially due to cultured fibroblasts being incorrectly calibrated [64]. In contrast, the “Skin-Blood” clock identified age acceleration in this progeroid disorder with higher sensitivity for these specific tissue types.

Cellular mechanisms, such as mitosis, will vary between tissues and cell types and contribute to changes observed in the epigenome. The DNA methylation state is re-established after replication, but errors occur in the fidelity of this process [65]. Therefore, a methylation-based clock of mitotic age will count cell-specific mitoses. Clocks capturing this process include “epiTOC,” which assesses increased DNA methylation in the promoters of Polycomb group target genes that are initially unmethylated in foetal tissues [31]. Replication-associated methylation accelerates in cancer and in pre-cancerous tissue due to carcinogen exposure. Mitotic age also drives the changes observed in the “Remethylated Window Model” with a loss of methylation in specific sparsely located CpGs that reside within partially methylated domains (PMD) [66], the megabase-scale late-replicating, lamina-associated hypomethylated blocks [67]. Hypomethylation accumulates with the number of cell divisions, due to relatively slow PMD remethylation, as well as a reduced efficiency in very low CpG density regions. Cellular damage or inflammatory factors that increase cell turnover led to increased methylation loss [66]. Senescence-related hypomethylation in these regions was also previously proposed to be affected by mislocalization of DNMT1 during the S phase [68]. Additionally, the influence of H3K36me3, which recruits DNMT3B to gene bodies [69], is an independent factor that can act to counter this decrease in DNA methylation. Of note, loss-of-function mutations in the H3K36 histone methyltransferase *NSD1* also accelerate the Horvath clock, thus implicating loss of maintenance of this chromatin mark in the DNA methylation changes detected by this clock as well [70].

The developmental stage of the cell can be distinctly observed in clock measures. Additionally, it has been shown that partial and full reprogramming with Yamanaka factors induces a steady decline and a complete resetting of the epigenetic age, respectively [24, 71, 72]. Of note, highly specific CpG methylation changes are observed with replicative senescence and aging in human mesenchymal stem cells [73]. In analogy to the age-associated DNA methylation patterns, the epigenetic modifications during replicative senescence are also reset during reprogramming into induced pluripotent stem cells [74]. In regard to the cellular niche, human hematopoietic cells do not exhibit epigenetic age acceleration upon transplantation into a faster aging species, the mouse [75]. Similarly, the DNA methylation age measurement from hematopoietic stem cell transfusion matches the age of the donor and not that of an older recipient [76, 77]. In other words, at the resolution of these data, the stem cell niche does not affect the epigenetic age.

Clock-like epigenetic alterations can have disease monitoring utility, even if they are not functional and are only passive changes reflective of underlying biology. However, interesting paradigms exist that may be indicative of broader and more widely applicable pathological mechanisms that connect epigenetic aging and disease. These include examples such as age-related promoter hypermethylation with *HAND2* and endometrial cancer [78], as well as Polycomb group target promoters and cancer [18]. Also, the stochastic process of epigenetic drift, which itself actually begins from early age [79, 80], may also play a disease-related role [81]. Age-related DNA methylation modifications disrupt DNA binding patterns of transcription factors (TFs) which regulate the activity of many genes, although currently without strong evidence of expression disruption [82]. Changes with aging have been observed in both the binding sites of the transcriptional repressor REST [83] and insulator CTCF [84]. However, instead of targeting housekeeping or essential genes, epigenetic drift changes tend to occur in the periphery of the protein-protein interactive network [85].

#### Current uncertainty

There is uncertainty around how the DNA methylation changes observed in clocks can accrue without replication, i.e., due to processes not related to cell replication (see Table 1). Most tissues are comprised of non- or slowly dividing cells, and different division rates occur in different tissues. Aging-related aberration of the epigenetic machinery is implicated in DNA methylation change over time. However, understanding this will require more detailed characterization of the levels of instability aside from DNA replication, and the extent to which this process is cell-, genetic-sequence-, or *cis* regulatory element-specific. Cumulative changes, as well as potentially stochastic factors, most likely influence mitotic rate and fidelity, repair, chromatin remodeling, and transcription. These aggregating mechanisms are not exclusive to each other and could be important in differing degrees at different loci or in different cell types. The Horvath clock is derived from a wide variety of tissue types and works across most of them (including sorted neurons) even though these cannot have the same history of cell division, so this clock is not measuring mitosis.

Another point of ambiguity to be acknowledged on the mechanistic side is that epigenetic interaction with TF binding and downstream gene expression is clearly not as simple as usually portrayed in classical models [86]. This complex and significant interrelation between DNA methylation and TFs in various functional elements, such as promoter CpG islands, enhancers, and CTCF loci [87], has revealed experimental evidence supporting not only a negative regulatory role but also in some cases a positive one [88–90].

The use of elastic net regression to construct DNA methylation clocks results in sparse but accurate estimators, with utility in predicting phenotypic outcomes. However, there is uncertainty and limitations in regard to their mechanistic insight, which may instead require more precise knowledge of the specific epigenomic modifiers and transcription factors involved. Age-related hypomethylated CpG sites are observed to be strongly enriched in enhancer-related loci, in both stem and differentiated cells [91, 92]. Decay through reduced active processes required for maintenance and DNMT and TET-related methylation turnover without cell division is however observed in exit from pluripotency [93]. It is possible that this process may be more prevalent at the most dynamic enhancer regions. Additionally, neuronal cells have revealed high post-mitotic expression of DNMTs and TETs [61, 94], and there are, furthermore, higher levels of 5-hydroxymethylcytosine in the brain [61].

#### Future experiments and recommendations

In the hunt for disease- and tissue-specific molecular markers of biological age, future experiments will require individual tissue-, disease-, and mechanism-specific analyses. While clinical utility may be derived from pan-tissue “clocks,” such as the Horvath clock, being incorporated into other broad measures such as “frailty” [95], other bespoke “clocks” may be constructed and employed for particular diseases. By directly focusing on clock-like modifications that represent disease-related variation in specific tissues, this may bring unique insights and pathophysiological measures. To determine where surrogate tissue can be used, it will be important to establish the level of concordance and discordance between specific tissues within and between individuals [96]. However, this is clearly difficult in more inaccessible tissues. These refined disease-specific clocks may bring improved level of molecular resolution, in evaluating age-related disease progression within an individual. Furthermore, there is potential clinical utility in using a measurement given in “years” to simply explain to patients the concept of complex organ-specific deterioration. Also, these data can be incorporated into prognostic or therapeutic algorithms.

For mechanistic insight, limited CpG clocks in surrogate tissues are unlikely to be highly discerning. Specific CpG tissue and disease-specific clocks will likely capture some aspect of the underlying mechanism but may have less traction than genome-wide or whole-genome approaches. These will have more power to give functional understanding of the drivers of clock-identified changes. However, these currently have their methodological limitations (discussed in “Challenge 4”). For full evaluation, this may require single-cell bisulfite sequencing (scBS-seq) studies, nanopore sequencing analysis, and further technological advancement to be fully realized (discussed in “Challenge 5”). These tissue- and disease-specific clocks in isolated cell types or cell type-aware analyses could enable greater insights into the molecular drivers of biological aging [97]. These processes need to be explored across all organ systems, to identify not only specific but also common mechanisms.

Computationally, biophysical models (mathematical simulations of biological systems) need to be explored, as machine learning methods, such as elastic net regression, only offer limited mechanistic insight due to their black box nature. For instance, such biophysical models are beginning to emerge for modeling mitotic age [98], for understanding patterns of DNA methylation heterogeneity in aging stem cell populations [99] and for understanding the relationship between age-associated patterns of DNA methylation and alterations in epigenomic regulators [100]. We envisage that future studies that build and improve on these models by explicitly incorporating the latest insights and understanding of epigenomic regulation may be necessary to dissect the inherent complexity of the epigenetic aging process.

As detailed above, this functional interrogation will require the construction of bespoke tissue-specific and disease-specific clocks. However, open-science protocols will need to be followed to maximize their use, as well as their further optimization and improvement. Therefore, the field must require that all clock algorithms are transparent and publicly available to support reproducibility and accelerate progress.

## Challenge 3

### Integration of epigenetics into large and diverse longitudinal population studies

#### Current knowledge

Longitudinal studies following individuals over the course of their lifetime have considerable advantages in evaluating causal risk factors in disease development. For DNA methylation clocks, these studies are also extremely valuable, as cross-sectional data cannot assess the dynamics of the clock-related changes and measurements over time within an individual. Thus, these analyses can evaluate the relative contributions to epigenetic clock variation, including consistent differences from the start of life, altered trajectories at particular life junctures, such as puberty, or gradual divergence over the entire life-course [9]. Furthermore, the predictive power of clocks for age-related disease can be directly assessed.

The vast majority of epigenetic clock studies to date have been conducted in adults and are cross-sectional in design. The few initial longitudinal analyses performed have seen little variation over epigenetic age acceleration assessment within the same decade [49], and within middle age, multiple clocks track closely [33]. One substantial meta-analysis of longitudinal data from Marioni et al. in five cohorts, comprising 4075 adult participants, identified a slower rate of increase of epigenetic age compared to chronological age with time, with both the Horvath and Hannum et al. clocks [101]. Also, there is a non-linear (logarithmic) pattern in the clock during teenage years [24, 79]. Therefore, the clock calculation by Horvath included a log-linear transformation for data points from younger individuals. When applied to longitudinal datasets, both the Horvath and Hannum et al. clocks show signs of an asymptote in later life, where chronological age increases at a faster rate than epigenetic estimated age (see Fig. 1c). Cross-sectional studies have also consistently shown strong biological sex differences, with men having greater positive age acceleration than women [102].

#### Current uncertainty

The non-linear rate of clock ticking and what may influence this is not precisely defined. The Horvath clock is seen to run the fastest during development, while during adulthood, linear associations are observed with clock years increasing at the same rate as chronological years, on average. The biological aging marker of epigenetic age acceleration assessed from birth shows minimal variation to adolescence and then increases with age [103] and is hypothesized to be influenced by developmental changes during childhood and adolescence [104].

The full extent of genetic influence on DNA methylation both within CpGs on the arrays and further beyond in the genome is still underappreciated [86, 105–108]. How significant and through which pathways genetic influences act on clock longitudinal dynamics is uncertain, but has begun to be explored [109], and further major meta-analyses are in progress. Twin studies estimate that the heritability of the epigenetic age acceleration is relatively high (*h*^2^ ~ 40%) [9]. This is even higher at younger age, implying, as we age, there is an increasingly environmental contribution to the age acceleration calculation [24]. Of note, a genome-wide association study for the Horvath clock calculated age acceleration identified five loci, including an intronic variant with unknown functional implications within the telomerase reverse transcriptase (*TERT*) gene [110]. It is still unclear how much deviation of epigenetic age from chronological age is driven by different rates in biological aging or genetically determined differences between individuals. Moreover, various threads of evidence indicate some epigenetic loci display increased variability with age, which may potentially be an important and distinct measure in capturing biological age [111]. This is also observed in longitudinal analysis, with a fraction of these age-varying CpGs identified to be under genetic influence [109, 112].

Further areas of uncertainty arose from the longitudinal meta-analysis of Marioni et al. [101]. Firstly, significant differences between the Horvath and Hannum et al. clocks were seen, as would be expected due to their differing tissue training sets. However, they further proposed that while some of the slowing of the clock rate in the elderly may be due to survivor bias, there may also be a plateau to epigenetic clock estimates. Intriguingly, a possible decline at late age has even been postulated [113].

Recently, Zhang et al. identified that correcting for blood cell type proportions attenuated the all-cause mortality associations with both the Horvath and Hannum et al. clocks [46]. This reduction was shown to be greater for clocks built from smaller training sets. Furthermore, the association with mortality lessened, even without cell type correction, with increased training set size. The biomarker power of specific clocks may be increased or decreased depending on the contribution of major cell proportions to the specific disease or trait being examined (see Table 1). Changes associated with immunological aging [114, 115] are clearly contributing to aspects of biological aging. However, more precision is required regarding how these manifest within individuals over the life-course, as well as which specific cell types drive distinct associations.

#### Future experiments and recommendations

Longitudinal studies enable the description of the phenotypic manifestations of aging within individuals [9]. Therefore, they are powerful for determining the predictive ability of the DNA methylation biomarkers of disease and outcomes in individuals. As these studies are generally designed with multiple, often frequent, biospecimen collection, those including early age and young adulthood will be able to query observed departures of predictors from chronologic age through this developmental period. Similarly, samples obtained over multiple timepoints from elderly subjects could address questions about slowing in epigenetically predicted age. The availability of multiple sources of DNA from various tissues over time would also facilitate robust multi-tissue age evaluations [9].

By identifying the best-designed studies, with appropriate tissues, physiological, functional, and molecular biomarkers, and disease monitoring, the relative disease predictive power of DNA methylation can be robustly assessed. Due to the expense, consensus on this investment will help its realization. There has been significant success in genetic studies using the rigorously phenotyped UK Biobank. This is not only through extremely powerful GWAS, but also collating this information into a calculated risk for specific common diseases with genome-wide polygenic risk scores (PRSs) [116] with potential clinical utility [117]. Many well-known cohorts have generated DNA methylation data [107, 118, 119], but, undoubtedly, it would be highly desirable to assay further powerful longitudinal studies in extremely large datasets of deeply phenotyped individuals. Understanding the dynamics of clock-estimated age will improve as more studies obtain repeated measures of DNA methylation. This could include an application of latent class analysis on categories such as early, late, or constant epigenetic age acceleration.

It would be beneficial to generate DNA methylation data at scale on one or more cohort studies that have (a) prospectively collected data and DNA samples, (b) deep phenotyping of age-related traits, (c) standard biochemical markers of aging-related decline, (d) repeated measures, and (e) genetic data. Given the derivation of human DNA methylation clocks from array-based data, the latest generation DNA methylation array (EPIC 850k) would be the pragmatic approach at the current time. However, the field is currently in transition between a reliance of array-based platforms that capture data on a small subset of CpG sites and sequence-based approaches. As noted later (in “Challenge 4”), the interrogation of a wider range of DNA methylation sites using sequence data will ultimately bring added insights into underlying mechanisms, but the cost of such an approach at scale and appropriate depth is currently prohibitive.

The interrelationship between genotype and DNA methylome clock changes could be robustly evaluated in any large epidemiological cohorts that are genotyped (for some, such as UK Biobank, a significant portion is soon to be fully sequenced). Therefore, chronological age estimation could potentially be improved after correcting for identified genetic effectors on this measure. More nuanced haplotypic integration of epigenetic and genetic variation will ultimately be required. It will also be possible to study the impact of how genetic variation can influence clocks driven by relevant causative factors, such as inflammation and immunological aging. The relationship between genotype and DNA methylation clock calculations can be exploited to gain insights into causal or mechanistic pathways. For example, in cohorts where both genotype and DNA methylation data are available, it would be feasible to apply a Mendelian randomization approach to appraise the causal impact of a potential determinant of clock-derived age [120, 121]. A hypothesis-free approach might include the application of LD score regression [122], which would use all genetic variants associated with clock age and compare these against all available GWAS data to search for traits that show common genetic architecture with DNA methylation clock age. This may shed light on potential pathways that influence aging.

There is considerable potential clinical utility in the incorporation of epigenetic data in disease prediction. Given the precision with which DNA methylation clock age can be estimated and evolving measures of biological, phenotype-, and disease-related age (e.g., PhenoAge [43], GrimAge [45]), it may be a useful tool in enhancing clinical prediction models of age-related disease incidence. Studies to date have assessed the combined contribution of genetic and epigenetic data to specific traits [123, 124] and have demonstrated the utility of using DNA methylation as an index of specific health-related exposures, notably smoking [125], to predict future disease risk [126]. This ability to use blood-derived DNA methylation as a systemic exposure measure will continue to be refined. Adding clock-derived measures of biological aging to such prediction models could bring enhanced sensitivity and specificity over and above that possible from self-reported measures of known risk factors. For example, cardiovascular risk could combine genetic PRS for this trait with GrimAge clock measures, which estimate cardiovascular disease-related risk, such as smoking pack-years, plasma beta-2 microglobulin, and other plasma proteins, and predicts time to coronary heart disease [45].

Regarding the issue of cell type deconvolution for clock association, this will be specific to the disease or trait being examined. Single-cell analysis, as detailed in “Challenge 5,” will also help pinpoint which cell type(s) is the most important and guide the use of cell type corrections in heterogeneous DNA samples for larger longitudinal and epidemiological studies.

Another very important issue is that all these genetic and epigenetic data and analyses are strongly biased toward populations of European ancestry and other populations are grossly under-represented. Further large-scale diverse longitudinal studies are imperative [127]. As mentioned, the extent of genetic influence is currently underestimated and will therefore need detailed analysis across multiple populations (see Table 1). Additionally, the unique advantages of monozygotic twin studies should also be borne in mind [105, 128], and the comparison of these non-genetically confounded studies with larger population findings may be illuminating. Another fascinating avenue to explore that may reveal novel insights are those contemporary populations worldwide that commonly exhibit extreme longevity, termed “blue zones” [129]. These regions include Nicoya in Costa Rica, Ikaria in Greece, a region of Sardinia in Italy, Okinawa in Japan, and Loma Linda in the USA [2].

## Challenge 4

### Genome-wide analyses of aging and exploration of additional epigenomic marks

#### Current knowledge

**Fig. 2:**
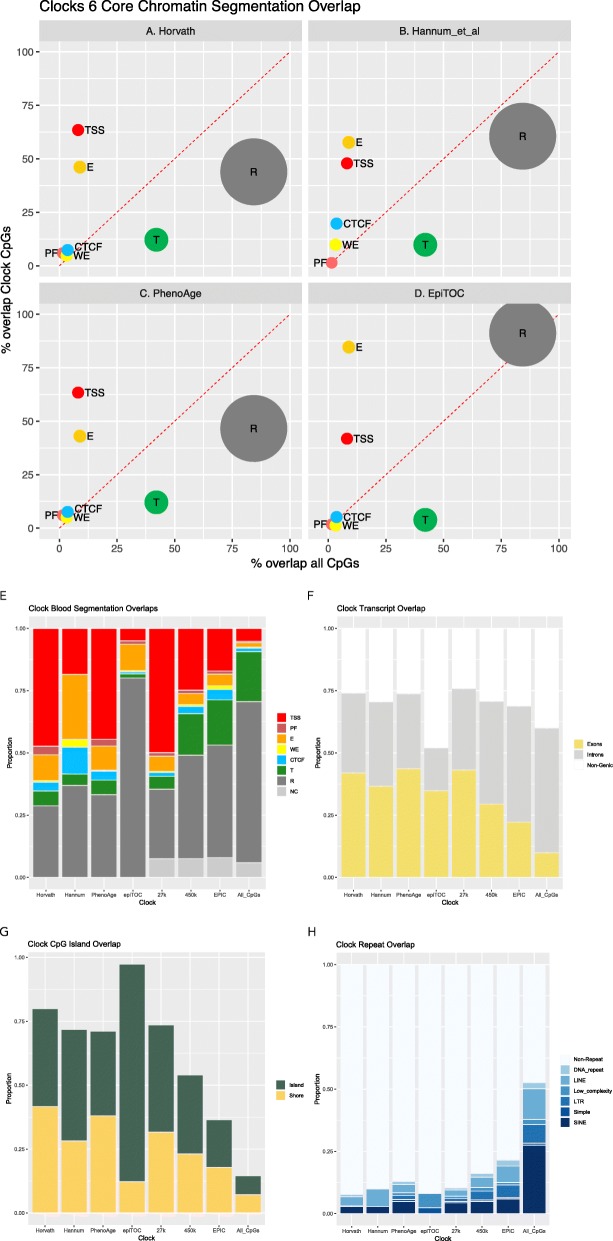
All clock probes are strongly biased to reside within active functional loci. This is due to their construction from promoter-focused arrays. Overlap of CpGs from four DNA methylation clocks with the six Core Encode Combined Chromatin Segmentation tracks [130] from ENCODE Analysis Data at UCSC. **a** Horvath clock [24]. **b** Hannum et al. clock [23]. **c** PhenoAge clock [43]. **d** epiTOC clock [31]. Location is assessed for overlap with the seven functional categories: PF (promoter flanking—light red), TSS (transcription start site and promoter region—red), CTCF (blue), WE (weak enhancer—yellow), E (enhancer—gold), T (transcribed region—green), and R (repressed—grey), from any of the six Core Encode cell types (Gm12878, H1hesc, Helas3, Hepg2, Huvec, K562). This percentage overlap is shown on the *y*-axis and is compared with the percentage overlap for all ~28 × 10^6^ CpGs in the human genome on the *x*-axis. Calculated via bedtools [131]. The size of the circle is proportional to the entire genome space for each functional category (~10^(genome size proportion)^). **e-h** Direct overlap comparison for four DNA methylation clocks (Horvath clock, Hannum et al. clock, PhenoAge clock, epiTOC clock) as well as Illumina array CpGs (27k, 450k, EPIC) and all genomic CpGs (far right bar) with: **e** the Combined Segmentation track for blood-derived tissue (GM12878) [130]. Functional segments are delineated as PF (promoter flanking), TSS (transcription start site and promoter region), CTCF, WE (weak enhancer), E (enhancer), T (transcribed region), and R (repressed). NC, not covered CpGs in this Combined Segmentation overlap; **f** Gencode [132] Exon and Transcripts; **g** UCSC [133]-defined CpG islands and shore regions (+/−2 kb); **h** Major repeat classes (UCSC RepeatMasker [133]), including DNA repeat elements (DNA_repeats), long interspersed nuclear elements (LINE), low complexity repeats and other rare repeat classes, long terminal repeat elements (LTR), simple repeats (microsatellites aka short tandem repeats), and short interspersed nuclear elements (SINE), of which ~63% are Alu elements

The initial DNA methylation clocks in humans were derived from the Illumina 27k or 450k DNA methylation arrays available at the time [21, 23–25]. Even the latest generation EPIC 850k only assesses ~850,000 sites, which is ~3% of all the CpG sites in the human genome. However, as aging changes are pervasive throughout the DNA methylome, these arrays easily capture age-related variation [23]. Only a small number of CpG sites are required in the clocks (i.e., Horvath: 353, Hannum et al.: 71, PhenoAge: 513, epiTOC: 385). The distribution of these selected CpGs, compared to genomic functional regions, can be seen in Fig. 2a–e and is clearly enriched for active loci (such as transcription start site/promoter regions) due to the available CpGs for selection present on the arrays. Within the DNA methylome itself, the likelihood of methylation variability at an individual CpG is associated with its surrounding CpG density [134], with intermediate density regions showing the most changeability through development, across tissue types, and in cancer [135, 136]. The proportion of variable sites (methylation change ~≥0.3) in normal conditions is estimated at 15–21% [137, 138] (see Fig. 2f and g for transcript as well as CpG island and shore distribution of clock probes, respectively).

The association between other DNA modifications and age in humans remains underexplored. Aging-related changes have been seen with hydroxymethylation (5hmC), even in blood, where this modification is infrequent [139]. More detailed analysis is required to assess whether this approaches the correlation identified for 5mC. Extensive chromatin changes with age are observed in model organisms (yeast, worms, flies, and mice) [140]. The co-ordinated nature of the epigenomic machinery through both chromatin modifications and DNA methylation [141, 142], as well as experimental evidence from chromatin regulators, such as DOTL1 [143], implies that aging chromatin clocks could also exist, although without the ease of assay and highly quantitative measurements that DNA modifications enable. Broad chromatin aging-related changes include loss of H3K9me3 within heterochromatin and redistribution of H3K4me3 and H3K27me3 in euchromatin [60]. Additionally, aging in a mouse brain leads to altered hippocampal chromatin plasticity with deregulation of histone H4 lysine 12 (H4K12) acetylation [144], as well as histone variant H2A.Z accumulation [145]. In human prefrontal cortices, aging-related changes were found in H3K9 acetylation (H3K9ac) associated with Alzheimer’s-related tau protein burden [146].

#### Current uncertainty

In regard to the DNA methylome, few sequencing-based studies have investigated regions beyond the limited CpGs profiled using array-based techniques [147–149]. The question is how much more information can we obtain by examining additional sites in the epigenome, what additional value this will bring, and where should we look? Sequencing-based studies will easily observe aging changes, but are expensive to perform, especially with whole-genome base-resolution techniques, and suffer from inconsistent regional coverage, making use of common clocks problematic. Furthermore, higher levels of coverage (~100X) than typically employed (5–30X) are actually required to call high-confidence differentially methylated positions (DMPs) [150]. Of note, Horvath’s pan-tissue clock exploits the 27k array and is highly accurate in predicting chronological age even without including the *ELOVL2* CpGs covered with the 450k array, which display age associations that are much stronger, and consistent across tissues, than any other CpG currently identified [46].

An additional question is whether we can glean new information about biological aging and not just a more accurate chronological clock. Repetitive elements, such as SINE (predominately *Alu* elements in humans), LINEs, and LTRs, of the genome could be responsive to environmental factors such as diet and stress, and as such are intriguing targets for further exploration [151]. Repetitive regions possess latent functionality [152, 153], however are robustly epigenetically repressed by DNA methylation [151, 154, 155]. Their hypomethylation with age could, therefore, be a significant underexplored pathogenic mechanism in non-malignant age-related disease. An early whole-genome bisulfite sequencing (WGBS) neonate versus centenarian comparison identified ~87% of the differentially methylated regions (DMRs) to be losing methylation with age [147]. Although changes in cell composition were not accounted for, it was still interesting to note that strong enrichment occurred in repetitive sequence, with ~18% of the age DMRs in *Alu* elements. The impact of this deterioration of their epigenetic state with age is however uncertain, and, furthermore, these repetitive elements are largely not represented on arrays for technical reasons. For example, only ~2.7% of the 450k array probes overlap *Alu* elements, and this small fraction is ~4x more likely to be from the recognized set of technically poor functioning probes [156]. Also, further unknown age-related pathology may exist with other repeat families, including the LTR12C subfamily of LTR repeats that possesses significant enhancer evidence in multiple tissues in humans [157]. While earlier targeted work was not strongly supportive of age-related promoter hypermethylation being due to adjacent or overlapping repeats [158–160], latent enhancer hypomethylation with age is another possibility [92]. Hypotheses proposed for the mechanism involved in DNA methylation loss within these loci include inadequate DNA methylation maintenance influenced by a lack of dietary substrates, such as those for *S*-adenosylmethionine (SAM) [81], or TET-mediated active DNA demethylation [161]. Interestingly, an assessment of the human DNA methylation clocks found that CpGs reducing in their methylation level with age, rather than those gaining methylation, were the most indicative of biological aging through their association with life expectancy [162].

A further key question is how interconnected across the entire genome are all these age-associated epigenetic changes and whether there are specific altered “hubs” which drive concerted changes at both the DNA and the histone modification level. If epigenetic drift could affect activity of one of these hubs, and likelihood of this happening may well increase with age, then drift could have a functional impact. The endpoint gain or loss of defined functional units, such as a specific enhancer, proposes that aging chromatin clocks could be more informative or sensitive. Furthermore, these epigenomic changes would be tractable for exploration in those model organisms that lack DNA methylation (e.g., yeast and *C. elegans*).

#### Future experiments and recommendations

There are several considerations for the next wave of studies into the biology of aging. First, analyzing a large number of samples is key (see “Challenge 3”), ideally in the 1000s. Over the course of the next few years, it is unlikely that any technology will be able to do this affordably at base resolution other than the current generation of Illumina EPIC 850k DNA methylation arrays. Therefore, at this point in time, large-scale population-based studies of the aging epigenome will continue with this robust array. Many of the additional sites on the platform may not provide any further independent information, although with the caveat that increased enhancer loci have been explicitly targeted on this array. This increased focus on enhancer CpGs potentially gives this array improved power to identify tissue-specific and disease-specific loci. Another possibility is a two-stage study design with the first step involving adequately powered WGBS to gain greater coverage to identify age-variable DNA methylation sites and regions, then designing a custom array that could be used at very low cost on very large numbers.

While the focus so far has been on DNA methylation, other DNA modifications, as well as known and currently unrecognized chromatin modifications, should be explored and may reveal exciting clock-like properties. Suggestively, the premature autosomal recessive aging disorder, Werner syndrome, while showing DNA methylation clock age acceleration [163], also has identified significant heterochromatin changes [164]. The optimum analysis of chromatin modifications requires fresh samples, but epigenome-wide association studies have been recently performed successfully with histone acetylation derived from post-mortem specimens [165]. These data can also be further integrated with DNA modification changes. Larger scale mass spectrometry quantitation of histone modifications could also be evaluated. Additional DNA modification analysis by oxidative BS-seq via array for 5hmC [166] should be further evaluated in aging, although this is still currently expensive to perform in large numbers. However, new methodologies, such as a non-destructive DNA deaminase [167], may help to propel these on.

Repetitive elements, where currently technically possible, may be sites for identifying aging-associated DNA modification in order to construct novel clocks, and these loci are clearly under-represented by arrays presently (see Fig. 2h). In this exploration, smaller scale whole-genome sequencing DNA methylome analyses should not be deterred. Analyzing repetitive elements by these methods is the only realistic option, and for the longer repeats, third-generation direct long-read sequencing may be required. Although they can measure modifications directly, given the sample numbers needed and the error rate in DNA methylation measurements for third-generation technology, such studies are 3–4 years away. However, current second-generation techniques may, despite technical challenges, reveal prospective loci that can then be robustly explored and validated through targeted amplicon BS-seq techniques via platforms such as Fluidigm Access Array [168] or as third-generation direct sequencing matures. Also, classic and novel chromatin marks should be scrutinized for unique clock-like signatures. An important future direction for functional exploration is to connect DNA modifications, histone post-translational marks, and transcriptional data into a single integrated aging model.

## Challenge 5

### Single-cell analysis of aging changes and disease

#### Current knowledge

Novel insights into aging-related biological changes will be identified by moving beyond the misleading homogeneity of bulk-cell-derived data to the heterogeneity of single-cell analysis [169]. Tissues age as both genetic and epigenetic mosaics, changing their cellular variation. This indicates that single-cell analysis will be necessary to accurately understand this process. This may pinpoint individual cell type age DMPs. Significant aging-related cell composition changes are observed in blood, which include a skew toward myeloid lineage-derived cells [170], diminishing immune competence and a shift from naive to memory T cells [171], and clonal competition [172]. These cell mixture changes occurring with age may be equally complex in other tissue types. In fact, single-cell techniques have recently recognized previously unknown pathologically relevant cell types, for example in the airway epithelium [173]. An important example in the context of aging is sporadic senescent cells that occur in aging tissues, with accumulating evidence that these cells may be a driver in deteriorating organ function [174].

A key question regarding the DNA methylation clock at the single-cell level is to what extent clock site changes are cell autonomous or conversely to what degree the clock is a cell ensemble phenomenon. As most of the age-related changes in DNA methylation are relatively small, it is perhaps more likely that for most clock (or even age-related) sites, the changes observed, even in relatively homogeneous cell populations, are not cell autonomous but rather cell population based. That is, they are occurring only in a subset of cells in a tissue. Single-cell data may bring answers to these questions, as well as insight into aging mechanisms beyond the current predictive power of DNA methylation clocks. Construction of a clock at the single-cell level is currently technically challenging primarily because of missing data in each individual cell. Computational techniques such as imputation may help with these absent values [175]. Nevertheless, findings from single-cell combined transcriptome and DNA methylome sequencing in mouse muscle stem cells have already shown specific context-dependent increases of cell-to-cell heterogeneity in methylation coupled with increased transcriptional heterogeneity, especially in stem cell niche genes [176]. Similarly, chromatin modification analyses in blood, while clearly indicating immune cell types, also identify an increase in cell-to-cell variability with age, or “epigenomic noise,” with particular increases in both H3K4me3 and H3K27me3 [177]. This variability is a molecular signature of immune cell aging and may be due to the rise of distinct clones. Twin analysis revealed the majority of the changes (70%) were non-heritable or environmentally driven, being in a similar range to the ~80% proposed for DNA methylation changes [107]. On the expression side, single-cell analysis in mice also identified an increase in variability with age, with greater cell-to-cell transcriptional volatility in CD4 T cells [178].

Bulk analysis of isolated cell populations can still give epigenetic insights and also hint further at what single-cell analysis will be able to refine with even further cell type or clonal resolution. For instance, in humans, bulk purified CD8 T cells show decreased naive and increased memory sub-fractions [114]. Additionally, ATAC-seq of aging naive cells demonstrated reduced promoter accessibility, especially for the DNA methylation-sensitive transcription factor NRF1 [114]. This is indicative of the integrated epigenomic and transcriptomic changes occurring during aging. On the genetic side, deep exome sequencing has identified age-related clonal hematopoiesis in blood [179] and positively selected clones show prevalent mutations in epigenome-modifying genes, *DNMT3A*, *TET2*, and *ASXL1* [179, 180]. These clones are pathogenically associated with not only hematological cancers [179, 180] but also non-cancer disease risks such as atherosclerosis [181].

#### Current uncertainty

Exploring clock-related changes at the single-cell level would determine the cell type drivers of tissue-specific clocks. However, at this point, a significant unknown is how discrete the epigenomic profile of distinct cell types are and how much of a continuum between cell types exists. Also, what changes are occurring prior to observable age-related changes, such as up-regulation of *trans*-acting TFs. How do the levels of DNMTs and TETs change, and what is their interaction with other substrates? With the initial Horvath clock, some organs had a larger error in estimation of chronological age [24], initially interpreted as faster biological aging rates, though now thought possibly due to the impact of hormones on tissues such as the breast [182]. While cell isolation techniques may enable robust and insightful studies due to larger sample sizes, their level of resolution may be limited by the methodologies employed and current knowledge of cell categorization [183].

Another area of uncertainty is pan-tissue aging changes. While there is strong indication of significant and perhaps unique outliers that exhibit aging changes across all tissue types [63], such as *ELOVL2* [46], analysis of the large number of tissue-specific changes with tissue-specific clocks will bring substantial insight (as discussed in “Challenge 2”). There is evidence for a significant level of shared age DMPs between certain tissues [62], and single-cell analysis will allow for more robust evaluation of both these observations to identify in which individual cells these occur.

Increasing levels of somatic genetic mutation with age are now recognized [184, 185], leading to distinct clones, with potential pathological involvement even in non-malignant age-related disease [186]. How this may impact in a cell- and disease-specific fashion throughout the epigenome and clock-related changes is another uncertainty in age-related pathophysiology, particularly if a mutational enrichment in epigenome-modifying genes is observed, as in cancer [187].

#### Future experiments and recommendations

**Fig. 3:**
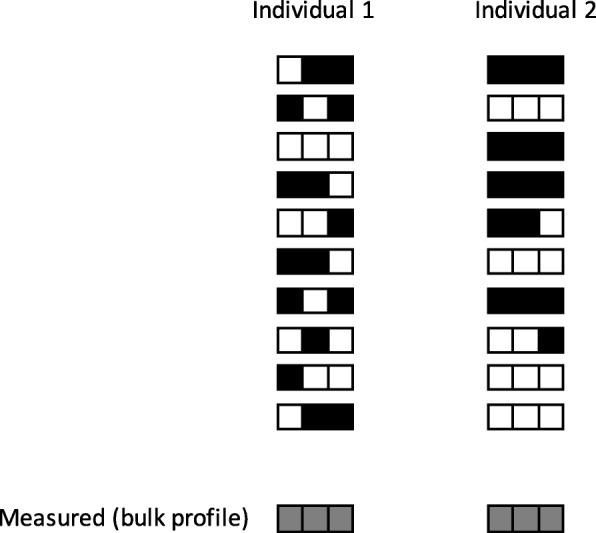
Single-cell analysis. Distinct cell variation in aging epigenetic clock changes may exist that would not be apparent in bulk comparison. Black and white squares represent methylated and unmethylated loci, respectively. Each row represents a single cell’s epigenome (represented as haploid for simplicity) with increased variability present in individual 2

Single-cell epigenomics will facilitate much more detailed exploration in both disease and age-related changes. While the technology is still evolving, successful datasets have already been produced and are able to give a level of resolution that is beyond previous expectations, with methods such as single-cell ATAC-seq and BS-seq [172]. This allows precise exploration of core epigenomic issues related to cell type heterogeneity and its tissue-specific modification with age. Single-cell analysis has the potential to reveal stronger cell type-specific changes that are currently diluted in signal in present results (see Fig. 3). This will include the identification of novel cell types, accumulation of senescent cells, and clonal competition that will manifest as epigenomic variation associated with age and age-related disease. Additionally, tissue-specific versus pan-tissue common aging findings can be further explored [62]. Single-cell analysis can also probe and more clearly evaluate and subcategorize phenomena observed with bulk analyses, such as increased DNA methylation variability with age [111].

This methodology will also allow dissection of cellular aging changes, such as the clock-like DNA methylation changes related to mitosis [31, 66]. Cell-specific age DMPs may indicate the binding footprints of specific TFs [87], enabling cell type-specific aging-related mechanisms to be defined. It is likely that with improved coverage of scBS-seq it will be possible to generate a single-cell clock; this may also be aided by further methodological or technical breakthroughs, including potentially single-cell multi-omics measurements [188]. Furthermore, modulation by experimental models may give further insight into the influence of particular substrates in observed aging changes. Data quality is steadily improving, although bulk-derived results are not obsolete and can complement and clarify single cell information due to issues such as non-specific results and reduced depth [172]. As these aging changes are so relevant to the function of cells and organs, we recommend that aging changes need to be clearly considered along, or in conjunction with the Human Cell Atlas initiatives where possible [189]. This will need attention in single-cell aging-related disease studies, and further complexity may exist through the intercellular environment via the influence of neighboring cells on cell aging trajectories.

The technology is rapidly developing, as illustrated by the recent sci-CAR (single-cell combinatorial indexing - chromatin accessibility and mRNA) assay, permitting the profiling of both chromatin accessibility and gene expression in thousands of single cells [190]. This and other maturing methodologies [191] will undoubtedly enable more precise understanding of age-related observations such as epigenomic drift. This should eventually enable single-cell epigenetic clocks giving a clearer indication of the functionality of these aging-related changes and guide further future experiments into the mechanism of disease.

## Challenge 6

### Generation of robust non-human data of aging

#### Current knowledge

Much of what we know about the biology of aging comes from studies on model organisms [6]. Taking advantage of all this knowledge, the development of DNA methylation clocks in these experimental systems brings with it an opportunity to advance our understanding of the role of the epigenome in aging. The Horvath clock initially demonstrated a strong correlation with age in our closest relatives: the chimpanzees and bonobos [24]. However, for the common laboratory mouse strains, only 1.6% of the EPIC array probes aligned to conserved CpG sites [192]. Age-related changes in the DNA methylome have been extensively analyzed in C57Bl/6 mice. Sziraki et al. described global remodeling of the mouse DNA methylome with age, reporting numerous global, region-specific, and site-specific features [193]. The associated genes and promoters were found to be enriched for pathways associated with aging, suggesting a fundamental relationship between the epigenome and the aging process. In addition, aging was accompanied by an increase in entropy, consistent with damage accumulation. Interestingly, the effects of this entropy varied for the sites that decreased, increased, and did not change DNA methylation levels with age. Some sites trailed behind, whereas some followed or even exceeded the entropy trajectory and altered the developmental DNA methylation pattern. The patterns found in certain genomic regions were also conserved between humans and mice, indicating common principles of functional DNA methylome modulation between species. As this study examined a whole range of mouse ages, it also detected accelerated changes in the DNA methylome in late life, which were not seen in studies with more limited age ranges [159, 194, 195]. Also, calorie restriction both shifted the overall methylation pattern and was accompanied by its gradual age-related remodeling. As in humans, with age, both highly and lowly methylated sites trended toward intermediate levels, and aging was accompanied by an accelerated increase in entropy [23, 41, 193].

A number of successful mouse sequencing-based DNA methylation aging estimators have been devised, including multi-tissue clocks [196–198], a liver clock [199], and a blood clock [200]. Field et al. have also recently described the clear strengths of mouse aging models [10]. One advantage of mouse models is the possibility of testing longevity interventions or modulators. For example, a multi-tissue clock was accelerated by high-fat diet [196], and the liver clock reported the effects of caloric restriction, dietary rapamycin, and Prop1^df/df^ dwarfism [199]. The blood-based clock revealed the impact of caloric restriction and dwarfism, as well as the influence of a whole-body knockout of the growth hormone receptor [200]. One of the most recent studies also carried out a comparative analysis of these mouse clocks, noting certain limitations between tissue and multi-tissue estimators [198]. A calorie restriction intervention led to significant changes in epigenetic age in mouse [200], and, furthermore, a 30% restriction in rhesus macaques led to an average DNA methylation age 7 years lower than chronological age [194]. In addition to the notion that the epigenetic clock sites may be variable in relation to metabolism, an overlap of age-related cytosine modifications with sites that exert epigenetic control of circadian machinery genes has been observed [201]. Wide-ranging mechanistic findings have also been drawn in mice, such as a *trans*-species experiment with an aneuploid mouse, which possesses a human chromosome 21 within its nucleus, revealing increased aging-related DNA-methylated changes, implying these are influenced by the cell’s nuclear environment [202].

The mouse has emerged as a significant model organism to study and quantify the epigenetic changes with age in a mechanistic way, e.g., the effects of longevity interventions on biological age were first demonstrated in mice. There is no doubt that further improvements in aging clocks in this species will lead to many discoveries both in the basis biology of aging and the discovery and validation of interventions that extend lifespan.

Additional models beyond the mouse may also be highly valuable, such as the naked mole rat with its extraordinary longevity compared to similar species [203]. Clocks have now been constructed in many animals, such as an estimator that can be used across both domestic dogs and wolves [204]. Variation identified within dog breeds with respect to aging is consistent with known lifespan differences [202]. The diverse range of wild animal clocks include, for instance, the humpback whale [205].

#### Current uncertainty

If DNA methylation clocks provide a readout of biological age, then it will be essential to determine its modifiability, whether or not therapeutic or lifestyle interventions will help to reverse it and gauge any impact on long-term health. The biological origins of the clock elicit a great deal of debate. Exploration will require experimentally and computationally deconvoluting the different processes of the clock into its constituent parts, where specific model organisms may have particular strengths. A broad variety of factors, beyond the clear role of genetics, are proposed in the dynamics of age-related methylation variation. These include inflammation, cell division, metabolic effects, cellular heterogeneity, diet, and a variety of other lifestyle factors, as well as stochastic effects. Regarding the possible involvement of metabolism, this is tightly intertwined with epigenetic regulation and nutrition specifically may modify DNA methylation [206], therefore pinpointing it as a potential significant mediator [207].

Further uncertainty arises from the obstacle that aging DNA methylation sites are only partially conserved among different mammalian species. Also, the inconsistent coverage from sequencing-based studies makes these less transferable, even across mouse experiments within the same tissue in the same strain. This technical issue with the reliance on sequencing, due to the lack of available commercial DNA methylation arrays in non-human species, reduces the utility of these published clocks.

#### Future experiments and recommendations

Construction of robust clocks in mammals and other vertebrates is likely to be highly informative for understanding aging. Mouse models have significant advantages due to their similar mammalian physiology, genomes, and epigenomes, but with a shorter lifespan, and ability to robustly control the animal environment. Most importantly, in contrast to human studies, direct genetic and pharmacological interventions can be more quickly tested in this species, although mouse aging experiments still take 3 years. So, for some questions, researchers can keep in mind the utility of short-lived vertebrate models with DNA methylation that are amenable to genetic manipulation, such as the killifish. Nevertheless, for consistent exploration of experimental clock modulation in mice, high-quality DNA methylome sequencing-based studies are needed. As mentioned in “Challenge 4,” depth for high-quality DMP calling in humans is estimated at 100X [150]. While this requirement will not be quite as stringent in isogenic mice, low-coverage studies should be avoided, due to their inherent lack of power. Additionally, clocks constructed with alternate statistical methods that are more resilient to significant inherent stochastic loss of data points are also required, as discussed by Zhang et al. [46]. Additionally, human age DMP or DMR findings, in conserved genomic loci, can be explored in the mouse for further mechanistic insight. However, as detailed above, this focus obviously does not preclude the value of other aging models. Domestic animals, for example the dog, taking advantage of its genetic architecture and known age-related breed disease susceptibilities [208], may be an informative model for non-invasive longitudinal monitoring. Horvath is currently designing a pan-species array, with a reduced set of common probes, to facilitate a common clock measure across a range of organisms [209]. The identification of the target genes of epigenomic regulatory elements [210], such as enhancers or insulators modified by aging-related changes, may be highly informative and enable subsequent functional exploration in humans.

The use of non-invasive DNA methylation clocks in conservation and ecology is another highly valuable aspect that should be taken full advantage of. This was discussed recently regarding a novel chimpanzee-specific age estimator [211] and also relating to a range of wild animals [212]. The knowledge of the age of individual animals is extremely beneficial in animal conservation, facilitating more accurate estimations of demographics such as population age structure and reproductive success [212]. The study of humpback whales clearly displays the utility of DNA methylation clocks, as while they have similar lifespans to humans, these whales have no reliable visual age indicators after 1 year of age [205].

## Challenge 7

### Inclusion of epigenetics within current genetic ethical and legal frameworks

#### Current knowledge

Epigenetics is implicated in many facets of aging, and DNA methylation clocks provide a molecular readout of aspects of this underlying complexity [9]. The high correlation with chronological age has led to their use in forensics [54–56], although further proposals, such as age estimation in refugees [57], have significant ethical issues. As yet no policies exist governing the reporting of epigenetic findings or biological age estimates, including those based on DNA methylation. This current shortfall has been recognized for some time [213, 214]. To stimulate the necessary discussions, the first pioneering epigenetic reports including age estimates have been issued to study participants of the Personal Genome Project UK [215]. A comprehensive framework on the Ethical, Legal, and Social Implications (ELSI) is still required to be developed and formulated [216, 217]. Further illustrating the future of epigenomic analysis, distinct personal and multi-timepoint longitudinal DNA methylome changes were recently reported in an individual in relation to their chronic disease state [218].

#### Current uncertainty

Ambiguity surrounds the ethics of measuring biological aging, or aging modification by changes to lifestyle, and how personal responsibilities can be balanced against the requirements of society (e.g., insurance, provision of health care). Producing an objective and accurate surrogate marker for biological aging will reignite an age-old discussion concerning how, and to what extent, individuals can be held accountable for their own behavior and the impact this has on their health.

As detailed in the preceding sections, while the DNA methylation clocks provide novel and intriguing avenues for the biological exploration of the aging process, there remains a significant lack of knowledge regarding the accuracy and robustness of this broad-scale age estimator (see Table 1). This is particularly concerning when it is now being proposed for legal age verification or life insurance calculations. We currently do not know the validity of the various different clock measures in an individual, across populations, with respect to rare and common genetic variation, across time, or under particular environmental conditions, exposures or physiological changes.

#### Future recommendations

Safeguarding autonomous decision-making and how to obtain adequate informed consent in advance of calculating an individual’s estimated biological age will require a complex framework, which has to be applicable for diverse circumstances. One set of measures will be required to cover obtaining consent from an individual who aims to attempt to decelerate “biological” aging by lifestyle change and wishes to use longitudinal analyses of a DNA methylation clock as a biofeedback marker. There will be a very different set of requirements for this personal monitoring, compared to more controversial societal and political issues, e.g., in the context of discrimination [219], socioeconomic circumstances, and migration. Using epigenetic data in an ELSI framework acceptable to all stakeholders will require transparent governance based on scientific accuracy, which will require significantly more rigorous scientific evaluation.

## Conclusion

With this perspective, we have detailed seven challenges alongside the experiments and recommendations to explore these (summarized in Table 2), which we hope will help to further the fascinating biological discoveries that have accompanied DNA methylation clocks. These detailed strengths, weaknesses, and areas of inquiry should stimulate new discussion and experimentation.

**Table 2:** Summary of recommendations arising from the challenges of studying DNA methylation clocks in the context of aging

Challenges and recommendations	
1. Delineation of the chronological and biological components of DNA methylation clocks	
• Quantify the accuracy and robustness of “forensic” age estimates from different DNA sources	
• Isolate pan-tissue “biological” aging changes for novel insights into aging	
2. Functional characterization of tissue-specific and disease-specific clocks	
• Refine tissue- and disease-specific clocks for disease-specific measures	
• Deeper understanding of the pathogenesis of specific age-related diseases	
• All published clock algorithms should be transparent and publicly available	
3. Integration of epigenetics into large and diverse longitudinal population studies	
• For predictive biomarkers of clinical utility	
• Understand the cause and consequences of clock measures and any rate change on aging-related disease and longevity	
4. Genome-wide analyses of aging and exploration of additional epigenomic marks	
• Identity novel and potentially more sensitive chronological or disease-specific clock-like mechanisms	
5. Single-cell analysis of aging changes and disease	
• Explore functionality of clock-like and other aging-related epigenetic changes	
• Define the components of tissue-specific changes	
6. Generation of robust non-human data of aging	
• Explore fundamental biology of aging using DNA methylation clocks in model organisms	
• Expand and standardize the application of DNA methylation clocks to test longevity interventions in mice	
7. Inclusion of epigenetics within current genetic ethical and legal frameworks	
• To educate and protect the public from misinformation and misuse	

The power of epigenomic analysis is clearly displayed by these precise aging-related changes. Detailed evaluation of DNA methylation clocks may reveal unique insights into the aging process itself, as well as act as a biomarker of biological age and inform on age-related common disease risk. While we have highlighted some caveats regarding the potential misuse of clocks, more detailed experiments should help to alleviate these. We have only begun to reap all of the insights that study of the epigenome will bring in deciphering physiology and pathology, and there is much promise for both improved human and animal health.
